# The National Pension Fund for Nurses and Hospital Officials

**Published:** 1888-01-28

**Authors:** 


### THE NATIONAL PENSION FUND FOR NURSES AND HOSPITAL OFFICIALS.

[All Inquiries and Communications should be addressed to the Acting Secretary, 38, Old Jewry, London, E.C.]

WE reprint the following from last week's *British Medical Journal*. It is important to point out that in actual working the premiums to be paid will be smaller, and the pensions larger than the *Journal* seems to anticipate. The calculations are based on the supposition that each nurse will be able to afford at least an eighth part of her income as premium; thus, a nurse with £25 salary can pay £3 2*s*. 6*d*. a-year, or rather less than one shilling and threepence per week. For this she will be entitled to sick pay when ill, and with bonuses, it is expected her retiring pension at 60 years of age, or earlier, will be not less than ten shillings per week for the remainder of life. In the following leading article commenting on the whole scheme, the *Journal* says:

"On various occasions during the last five or six years we have directed attention to the defenceless position of women engaged in the work of sick nursing when incapacitated by illness or age from continuing in the exercise of their vocation. It was pointed out that with the modern development of sick-nursing into a profession, the old pension system which existed at a few hospitals was not adequate to meet the necessities of the new conditions under which a nurse no longer remained attached to one hospital all her life, but after receiving her technical education most commonly obtained a situation in another hospital not provided with a training school, or became attached to one of the institutions which provide nurses for private cases. The great difficulty in making provision for pensions lay in these newly-formed migratory habits, and in the frequency with which the occupation was abandoned after a few years' trial.

"The formation of The Hospitals Association seemed to promise an opportunity of testing the feasibility of establishing a provident pension fund for nurses, and it is with sincere satisfaction that we notice the preliminary difficulties which necessarily surround the inception of such a scheme have been overcome. Four merchants and bankers of the City of London —Messrs. Gibbs, Hambro, J. S. Morgan, and Rothschild— have undertaken to provide the sum of £20,000, which must, in order to meet the provisions of the Friendly Societies Act, be invested in the name of trustees before such a scheme as that of the 'National Pension Fund for Nurses and Hospital Officials' can commence work upon the scale which is proposed.

"Great credit is due to Mr. H. C. Burdett for the labour which he has bestowed upon the preparation of the scheme, the success of which seems now assured, if only the persons it is primarily proposed to benefit—that is, the nurses themselves—will come forward and show that they are in earnest. The scheme is founded on the provident principle, and the main resource of the fund will, it is expected, be the contributions of the nurses and other officials serving in hospitals. It is, we are glad to see, intended to make the fund self-supporting, and to fix the premiums and pensions to be paid at sums calculated by the actuary as safe. Contributions from the general public or from hospitals would be regarded in the light of a fund to provide bonuses. Contributors to the bonus fund will, we are informed, constitute the members of the society, and will elect half the council, the other half being elected by the nurses and other policy holders.

"One of the difficulties of the fund will be the relative frequency with which nurses will still continue to leave that occupation in order to marry, or for some other reasons which now operate to produce the very rapid thinning of the ranks which is always going on. This is to be met by providing a special scale of pensions for persons who reserve the right to require repayment at any time of all sums paid in to the fund.

"The fund, too, will operate to diminish, though never altogether to do away with, these frequent retirements. In many cases a nurse only gives up her occupation because she has no prospect of making any provision for old age; the consciousness that this can in future be done by the exercise of a moderate amount of self-denial during the working years of life ought to have an important effect in retaining women who have once embarked in the career. If, as it is only reasonable to expect, the public contribute freely to the bonus fund, that will afford an additional inducement to a nurse to remain true to her calling.

"Another difficulty which must be met is the relative rarity of habits of providence among women; they have had hitherto no organised help in this direction, and no societies which afford a standard, so that they are apt to think the premium high and the pensions small. It is not necessary to discuss the rates which it has been tentatively proposed to fix; these must be worked out on ordinary actuarial principles. This has been carefully studied by competent authorities, and it appears to be most desirable that as little delay as possible should occur in publishing them; sixty-five shillings a-year will undoubtedly appear a large sum to nurses of 25 drawing £25 a-year in wages; but when persons of business experience, whose advice they may be able to obtain, are satisfied that the rates are on a sound basis, a great step will have been taken towards gaining the confidence of the nurses. Some kind of test may have to be applied to applicants in order to ensure that only those who can be properly described as trained nurses are admitted as subscribers to the fund. The class which is to be included under the term 'hospital officials' must also be defined; difficulties will, of course, arise on this head, but they are only such as men of wisdom are accustomed to deal with. It would obviously be desirable to limit the fund to persons holding positions of responsibility and trust.

"The object of the scheme is good, and its main features are such as will commend it to persons acquainted with the wants of the workers in our hospitals; great care, patience, knowledge, and skill will, however, be required in working out the final details of the scheme; such qualities we may expect to find in those who are now engaged in its elaboration.

"The fact that the leading officials and treasurers and house-governors of so many great hospitals are showing an active interest in working out the scheme, and that it has secured the munificent support of merchant princes, proverbially as prudent as they are generous in a good cause, afford the highest guarantees of real and permanent success. The interest shown in the scheme by Sir Andrew Clark and Dr. Bristowe is only an earnest of that good will which the whole medical profession may with certainty be expected to show towards a class of persons—hospital officials and nurses—with whom they are intimately and daily associated in the great work of charity, in their daily lives, whose admirable qualities they appreciate, and whose welfare they may be counted upon to aid and promote by their influence and advice, and, when necessary, their active co-operation."

